# Myxoid solitary fibrous tumor with rapid growth due to increased mucinous components: a case report

**DOI:** 10.1186/s44215-023-00081-y

**Published:** 2023-08-01

**Authors:** Kaoru Kondo, Toshio Shiotani, Shinichi Furukawa, Mototsugu Watanabe, Kazuhiko Kataoka

**Affiliations:** Department of Thoracic Surgery, National Hospital Organization Iwakuni Clinical Center, 1-1-1 Atago-Machi, Iwakuni-City, Yamaguchi 740-8510 Japan

**Keywords:** Myxoid solitary fibrous tumor, Solitary fibrous tumor, Video-assisted thoracic surgery

## Abstract

**Background:**

Myxoid solitary fibrous tumors are defined as solitary fibrous tumors with ≥ 50% mucinous components. As they are a rare type of pleural tumor, no reports on their rapid growth before surgery exist.

**Case presentation:**

Herein, we report the case of a 63-year-old male patient with a myxoid solitary fibrous tumor. The tumor had grown rapidly from 27 to 50 mm over 6 months, and a contrast-enhancing area was observed in approximately one-quarter of the tumor on computed tomography. The tumor was located in the parietal pleura at the ventral part of the left fourth intercostal space without adhesion or invasion into surrounding organs. It was completely resected via video-assisted thoracic surgery. Based on histopathological and immunohistochemical findings, the tumor was identified as a myxoid solitary fibrous tumor. The patient was discharged on postoperative day 2 and has had recurrence-free survival for 6 months postoperatively.

**Conclusions:**

To the best of our knowledge, this is the first case to report the rapid growth of myxoid solitary fibrous tumor despite its predominantly benign nature. Myxoid solitary fibrous tumors should be considered in the differential diagnosis of rapidly growing preoperative tumors.

## Background

Solitary fibrous tumors (SFTs), particularly thoracic SFTs, are stromal tumors that predominantly originate from the visceral pleura. Although most SFTs lack mucinous components, myxoid SFTs are those with myxoid changes in ≥ 50% of the tumor volume [1]. However, given the limited literature on myxoid SFTs [1], including reports describing tumor progression, their characteristics remain unclear. Herein, we report a case of myxoid SFT with rapid growth over a relatively short period.

## Case presentation

**Fig. 1:**
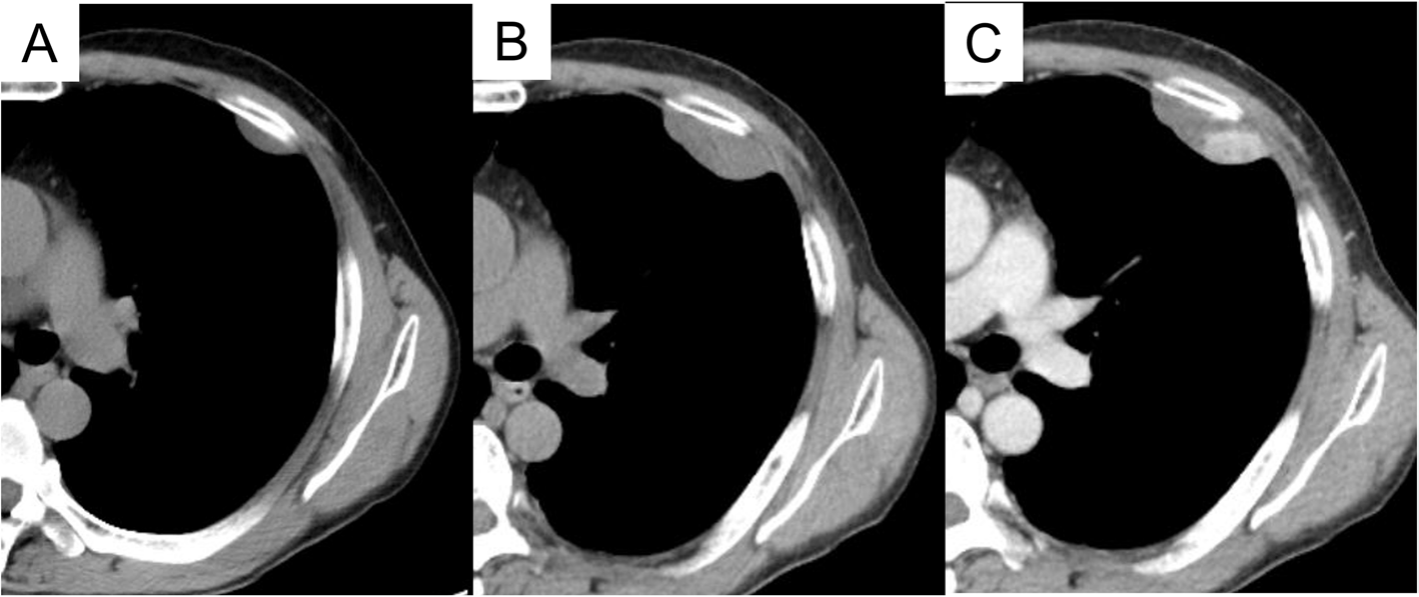
Chest computed tomography (CT) showing the tumor on the left chest wall in the thoracic cavity. On CT, the tumor size was 27 mm at the annual medical checkup (**A**) and had increased to 50 mm six months after the checkup (**B**). Contrast-enhanced CT showed a contrast-enhancing area within the tumor at the same time (**C**)

During an annual checkup, a 63-year-old male patient presented with an abnormal shadow in the left lung field on a chest radiograph. Computed tomography (CT) revealed a 27 mm tumor in the left chest wall (Fig. 1A). Six months after the initial checkup, the tumor had rapidly grown from 27 to 50 mm on CT (Fig. 1B), and a contrast-enhancing area was observed in approximately a quarter of the tumor on contrast-enhanced CT (CECT) (Fig. 1C). Blood investigations showed no abnormal findings, including tumor markers. Differential diagnosis of the rapidly enlarging tumor on the left chest wall was considered to include malignant pleural mesothelioma, malignant peripheral nerve sheath tumor (MPNST), SFT, or schwannoma. Given the absence of obvious unresectable findings, such as malignant pleural effusion or pleural dissemination in the thoracic cavity, and the rapid growth of the tumor over 6 months, we decided to perform resection of the chest tumor via video-assisted thoracic surgery.

**Fig. 2:**
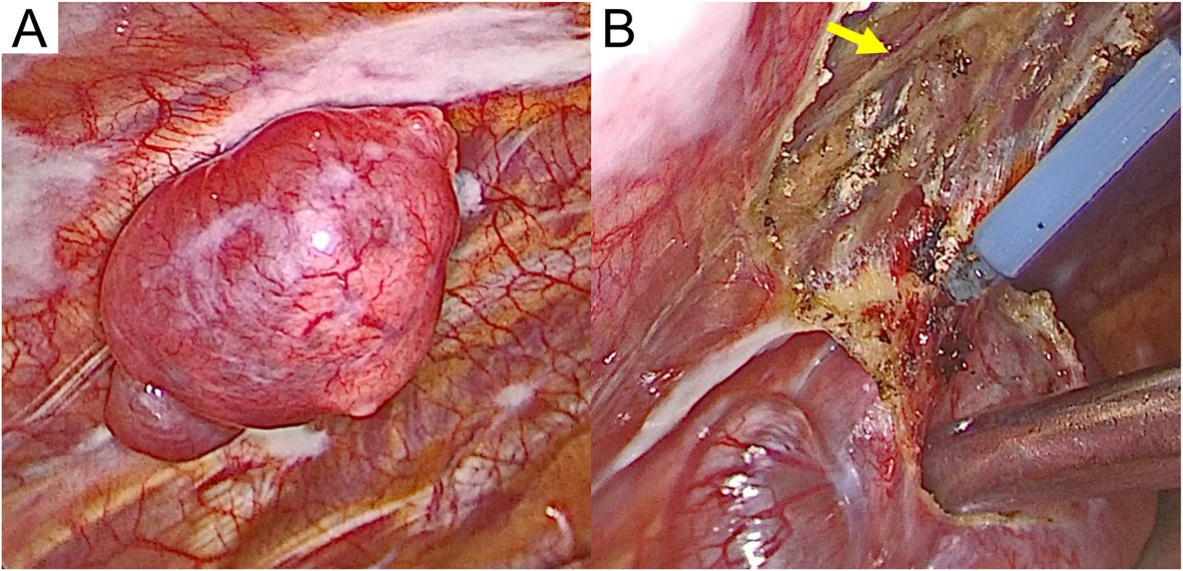
Surgical findings of the tumor. The tumor, which consisted predominantly of cysts with mucus (**A**), originated not from an intercostal nerve (arrow) but from the parietal pleura (**B**)

The tumor mainly consisted of mucus-containing cysts and a few nodules, and its surface was well-defined and smooth (Fig. 2A). It was located in the parietal pleura at the ventral part of the left fourth intercostal space, without adhesion to the left upper lobe of the lung and originated from the parietal pleura rather than from an intercostal nerve (Fig. 2B). Additionally, no evidence of pleural effusion and dissemination was observed in the left thoracic cavity. Based on these intraoperative findings, the tumor was considered benign. Hence, it was completely resected, including the partial pleura at the base of the tumor, and intraoperative consultation pathology was not employed.

**Fig. 3:**
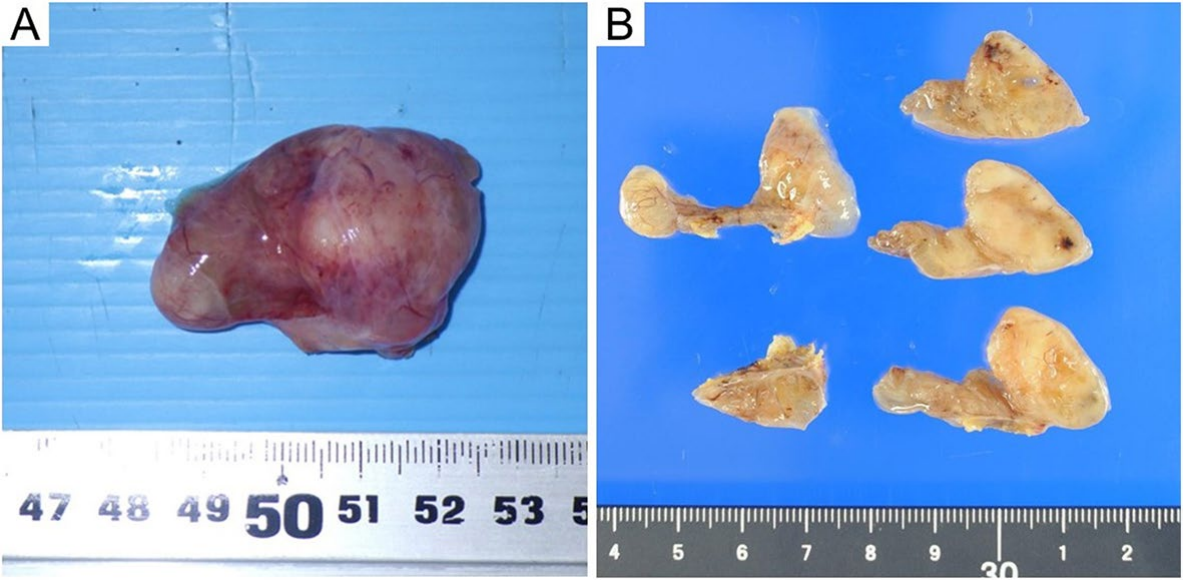
Gross findings of the tumor. The surface of the tumor was smooth, and a white lesion with elastic softness was observed in the nodular area of the tumor (**A**). On the cut surface of the tumor, there was a mixed distribution of pale-yellow and mucinous areas (**B**)

**Fig. 4:**
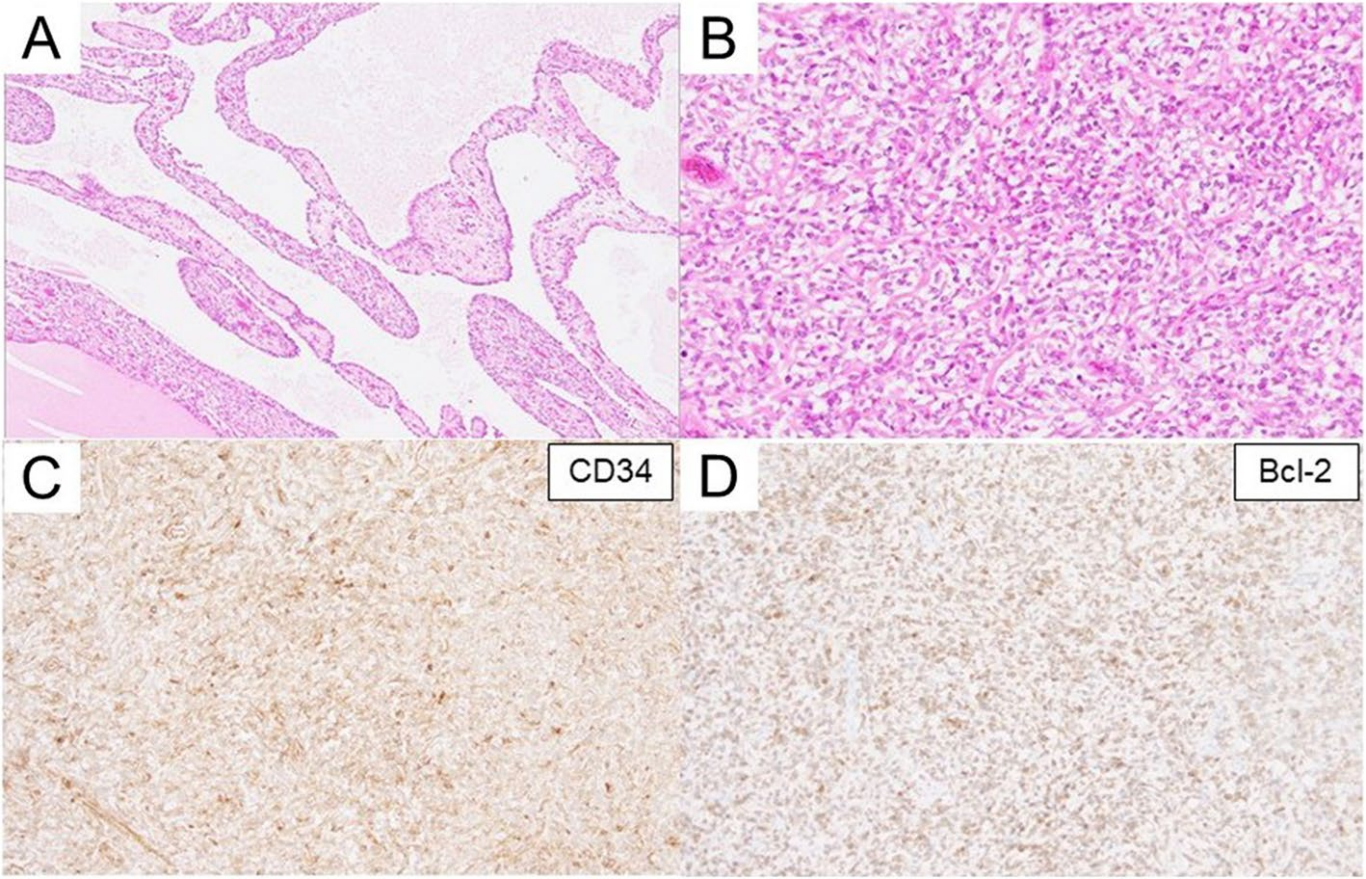
Tumor histopathological findings on hematoxylin and eosin staining (**A** and **B**) and immunohistochemical examination (**C** and **D**). Multiple mucinous cystic components were observed within the tumor (**A**). The tumor had patternless short spindle-shaped cells with hyper- and hypocellular areas (**B**). The tumor was both CD34 and Bcl-2 positive (**C** and **D**). (**A** magnification × 40; **B**, **C** and **D** magnification × 100)

The tumor had a smooth surface, and the nodular area of the tumor was a white lesion with elastic softness (Fig. 3A). The cut surface of the tumor had a mixed distribution of pale-yellow and mucinous areas (Fig. 3B). Histological examination with hematoxylin and eosin staining showed that the tumor contained multiple cysts with prominent mucus components (Fig. 4A) and had patternless, short, spindle-shaped cells and hyper- and hypocellular areas (Fig. 4B). Furthermore, immunohistochemical examination revealed that the tumor cells were positive for both CD34 and Bcl-2 (Fig. 3C and D), and negative for AE1/AE3, EMA, CD99, α-SMA, Calretinin, and D2-40. The percentage of MIB-1-positive tumor cells was approximately 2%. Based on these results, the tumor was diagnosed as a myxoid SFT. The postoperative course was uneventful, and the patient was discharged on postoperative day 2. Recurrence of SFT with mucinous production was not observed up to 6 months postoperatively.

## Discussion and conclusions

Myxoid SFTs are defined as SFTs exhibiting ≥ 50% mucinous changes [1]. Regarding the prognosis of myxoid SFT, no cases of local recurrence or high-grade tumor with distant metastasis have been reported [1]. Although myxoid SFTs are considered to have a good prognosis, their pathogenesis is unknown. To the best of our knowledge, this is the first case to report the rapid growth of myxoid SFT despite its predominantly benign nature.

Myxoid SFTs should be considered, along with MPNSTs and other SFTs, in the differential diagnosis of fast-growing pleural tumors with mucinous components [2, 3]. The rapid growth of MPNSTs can be induced by an increase in tumor cell components, as evidenced by the mottled tumor interior of enlarged MPNSTs on CECT [3]. In addition, some SFTs can also exhibit similar growth speeds due to internal degeneration, necrosis, or hemorrhage of the tumor, in which case, the contrast within the tumor is often nonuniform on CECT [4, 5]. In the present case, the myxoid SFT was enlarged, similar to MPNST. However, its rapid growth may have occurred due to an increase in the mucinous components rather than in the tumor cell components because CECT showed a clear boundary between the small and large non-contrast-enhanced areas. Therefore, a rapidly growing pleural tumor with a large non-contrast-enhanced area on CECT may be a characteristic finding of myxoid SFT.

Given that imaging may be an essential tool in the differential diagnosis of rapidly growing pleural tumors, magnetic resonance imaging (MRI) and fluorodeoxyglucose-positron emission tomography combined with computed tomography (FDG-PET/CT) may be helpful and further contribute to the diagnosis. MRI can help distinguish tumors from mediastinal and vascular lesions, confirm invasiveness, such as local invasion of the chest wall or diaphragm, and discriminate between solid and fluid tumor components [6]. The sensitivity and specificity for distinguishing benign lesions from malignant pleural diseases have been reported to be 92.8% and 94.1%, respectively, using diffusion-weighted imaging and dynamic contrast-enhanced sequences [6]. Like MRI, FDG-PET/CT may be useful for differentiating benign lesions from malignant pleural diseases, including malignant pleural mesothelioma, MPNST, and metastatic pleural tumors, with good results in both sensitivity and specificity [7, 8]. Unfortunately, in this case, MRI and FDG-PET/CT were not performed before complete resection because we decided to proceed with the surgery to diagnose and treat the tumor, whether the rapidly growing tumor was malignant or not. Nonetheless, FDG-PET/CT can evaluate tumor characteristics, while MRI can evaluate tumor characteristics and the degree of tumor involvement; hence, they should be performed preoperatively in similar future cases.

In conclusion, this case was a rapidly growing myxoid SFT, which may have been caused by an increase in the mucinous components. Further reports of cases are warranted for the detailed characterization of myxoid SFTs.

## Data Availability

Not applicable.

## Abbreviations

CECT: Contrast-enhanced CT
CT: Computed tomography
FDG-PET/CT: Fluorodeoxyglucose-positron emission tomography combined with computed tomography
MPNST: Malignant peripheral nerve sheath tumors
MRI: Magnetic resonance imaging
SFTs: Solitary fibrous tumors
